# Factors Contributing to Patients' Preferences for Primary Health Care Institutions in China: A Qualitative Study

**DOI:** 10.3389/fpubh.2020.00414

**Published:** 2020-08-18

**Authors:** Weiwei Zhang, Carolina Oi Lam Ung, Guohua Lin, Jie Liu, Wenjun Li, Hao Hu, Xiaoyu Xi

**Affiliations:** ^1^The Research Center of National Drug Policy & Ecosystem, China Pharmaceutical University, Nanjing, China; ^2^State Key Laboratory of Quality Research in Chinese Medicine, Institute of Chinese Medical Sciences, University of Macau, Macao, China

**Keywords:** patient, preference, primary health care, qualitative, China

## Abstract

**Background:** Primary health care (PHC) is a key component of the health care system in many countries. In China, however, PHC institutions are less preferred by patients, leading to the underuse of PHC services. Factors affecting patients' preferences for PHC institutions in China remain unreported in the current literature, which was first explored in this study.

**Method:** A qualitative interview study was conducted in Nanjing, Jiangsu Province of China. A semi-structured interview guide was applied to ask patients' opinions regarding the PHC institutions in China. Qualitative data analysis was conducted using the thematic framework approach.

**Results:** A total of 142 participants were interviewed. Three themes and their sub-themes emerged from the study: (1) attributes of PHC services, including accessibility of primary healthcare services, consultation time, drug cost, continuity of care, referral system, opening hours, waiting time, and drug accessibility; (2) attributes of PHC doctors' workforce, including doctors' attitude, competence, and accessibility; (3) attributes of PHC facility infrastructure, including basic facilities, diagnostic facilities and department settings. It was identified that some attributes of PHC services had positive impacts on participants' preferences for PHC institutions, while the same attributes of PHC doctors were the opposite.

**Conclusion:** There are three major factors that contribute to patients' preferences for PHC institutions in China. Policy interventions to improve doctors' workforce and facility infrastructure of PHC institutions are needed to promote patients' preferences for PHC.

## Introduction

Primary health care (PHC) is the essential health care universally accessible to individuals and families in the community, and PHC services are affordable to the community and the country through the full participation of healthcare providers based on practical, scientifically sound and socially acceptable methods and technologies (1). PHC is one of the key elements in the health care system as well as the first-contact between individuals and the health system (2), which provides various types of medical services, such as the diagnosis and treatment of chronic diseases, the health education, etc. By performing those services, PHCs cover most health problems of the residents at the scale of community. Essentially, PHC is performed based on patients' needs, so understanding the patient's requirements and preferences of PHC can help the policymakers and the health care providers identify the priority of different kinds of primary health services and improve the patients' compliance of medication (3). Governments around the world have tried to promote the utilization of PHC through various health policies for the effective allocation of medical resources, the equity of PHC services, and the improvement of residents' health.

In China, PHC system plays an important role in public health with a growing demand for health by the public. Therefore, in 2009, the Chinese government launched the new round of healthcare reform that a comprehensive PHC system was proposed to be reinforced to improve the residents' accessibility to PHC (4). Since then, the government has issued a series of policies on the re-construction and development of PHC system to enhance the provision of PHC services, such as increasing the government's investment, expanding the coverage of the universal health insurance, implementing the national basic public health service program, etc. One of the main strategies is the establishment of the National Hierarchical Medical System (NHMS). It is a triage system based on close collaboration among health care institutions of different levels intended to achieve the optimal outcome by elaborating on their roles and functions in the health care system (5). This three-tier health care system consists of PHC institutions, secondary hospitals, and tertiary hospitals. PHC institutions mainly provide basic preventive care and medical services through community health care institutions in urban areas, township health centers, and village clinics in rural areas (6). After the implementation of the relevant policies, PHC institutions have increased significantly in quantity. Studies showed that the number of PHC institutions in China was 699,000 in 2009, which accounted for 76.2% of the total number of health care institutions (7). By 2016, the number had reached 926,500, accounting for 94.2% of the health care institutions and had basically achieved the goal of comprehensive coverage of PHC (8, 9). In addition, the government's financial investment in PHC institutions has increased. For example, the proportion of PHC's financial subsidy in 2016 reached 43.8% of the total PHC revenue (10), which indicated the Chinese government's expectation to improve the service capacity of PHC institutions by increasing the financial investment in the public sector.

However, PHC service utilization of Chinese residents has not reached the expectation since the implementation of the relevant policies. The number of outpatient consultations in PHC has not increased significantly, and patients still seek medical care at the secondary or tertiary hospitals instead of PHC institutions as their first visit (11), leading to excessive demand and burden on the secondary and tertiary hospitals and the underutilization of PHC institutions (12). Only 55.19% of the patients visited PHC in 2018, China (13), suggesting a considerable wastage of high-cost medical resources in the secondary and tertiary hospitals, which can easily result in prolonged waiting time for patients and excessive workloads for doctors and other healthcare providers (14). In order to promote the utilization of PHC services, it is necessary to explore the factors that impact patients' choice of healthcare institutions among the Chinese population.

Literature review shows that the factors influencing patients' choice of health care institutions mainly include patients' personal preferences, social and cultural factors, sociodemographic characteristics, psychological factors, etc. Preferences are defined as ideas that individuals have about what they feel ought to be done, that is, normative expectations (15). Lega et al. in 2008 used a qualitative approach to explore the reasons why non-urgent patients preferred to choose an emergency department rather than a PHC institution (16). The results indicated that this preference was determined by the higher trust, convenience, and satisfaction with previous visits experience. A study by Northington in 2005 showed that patients' choice of health care institutions was associated with a lack of medical insurance in the United States (17). Berkelmans et al. in 2010 explored elderly individuals' perception of preferences for PHC and issues related to the non-medical attributes of PHC (18). The findings suggested that general practitioners' knowledges and attitudes might affect elderly individuals' preferences for PHC.

While some studies have investigated patients' preference for PHC in many countries, the health care systems of those countries are different from that in China. For example, in the UK, whether patients prefer primary care institutions or not, they have to visit general practitioners (GPs) in order to get a referral to a higher-level health care institution whenever necessary due to a strict referral system. Unlike the UK, Chinese patients have the right to choose different levels of health care institutions regardless of their conditions, so patients' preferences have a direct impact on the utilization of health care services across different levels of the healthcare system.

So far, no research has been conducted to explore patients' preference for PHC institutions in China. Wang et al. in 2010 showed that the determinants of patients' health-seeking behavior were affected by the type and severity of the disease, the hospital's medical technology, and cultures (19). Zhang et al. in 2010 suggested that factors influencing patients' choice of health care institutions included the distance between the medical institution and the patient's residence, the patient's economic status, and the severity of the disease (20). Yu et al. in 2017 indicated that although the medical costs in the secondary and tertiary hospitals were higher than that in PHC institutions in China, the health care institution with a more advanced hospital setting was more preferred by the patients. However, the research on patients' preferences for PHC is rarely mentioned or has not been explored thoroughly in China.

Thus, this study aimed to explore the factors contributing to patients' preferences for PHC institutions in China. In this study, we focused on the perceptions expressed by patients themselves, which provided a new perspective to help policymakers develop further policies and regulations that optimize the use of PHC services in China. In addition, it is expected that the findings can provide references to promote PHC utilization in other countries where the effective utilization of healthcare resources remains a challenge.

## Methods

### Research Design

This was a qualitative exploratory study. A qualitative interviewing approach was used to effectively assess residents' preferences for PHC services (21). This study focused on the patients themselves because they were the ultimate decision-maker of their health care choice.

### Research Ethics

The approval to conduct this study was granted by the Ethics Committee of the China Pharmaceutical University (Project number: CPU2018016).

### Interviewee Recruitment

The interview study was carried out in Nanjing City, Jiangsu Province, from January to June 2018. Nanjing is the capital of Jiangsu Province with a large population and relatively developed economy among cities in China. It has a relatively well-constructed primary health system consisting of about 150 PHC institutions, which have provided service for the entire urban population since 2018. However, the qualities of PHC services provided in different districts vary remarkably. Therefore, the qualitative study in Nanjing City could provide rich information about contributing factors of patients' preferences for PHC service, which increased the transferability of the findings from this study.

To ensure collect comprehensive information reflecting different situations of PHCs, 2 PHCs were selected by convenience sampling from each of the 11 districts in Nanjing. Eventually, 21 PHCs were visited, and 1 PHC dropped from the sample, covering about 14% of the PHCs in Nanjing. In each selected PHC institution, potential participants were approached on 1 or 2 random weekday(s) and 1 or 2 random weekend day(s) within 6 months, and within each day, they were approached evenly in the morning, the noon and the afternoon, to improve the variety of the participants. The sample was adjusted for even distribution of sex and age (young male, young female, middle-aged male, middle-aged female, elder male, elder female) by visually observing the potential participants. Eight participants were designed to be recruited in each selected PHC. The inclusion criteria were that the resident (1) performed at least 1 visit both at a PHC institution and a secondary/tertiary hospital in the past 12 months; (2) had the ability to express himself/herself. Eligible participants who agreed to participate signed a written informed & publication consent prior to the start of the interview.

### Data Collection

Semi-structured interview was conducted for each interview (30–60 min duration). There were two researchers in each interview who were familiar with the research design and background, understood PHC services, and were experienced in qualitative research methods. One researcher performed the interview and the other recorded the responses and controlled the paces and quality of the interview.

An interview was performed when the participation of a participant was confirmed. During the days for interviews, random participants who had finished their PHC visits and prepared to leave the PHC were provided with the purpose of this study, the processes of the interview, and the inclusion criteria of the participants. Those who met the inclusion criteria were willing to participate and had signed the informed & publication consent were the eventual interviewees.

During the face-to-face interview, we explored residents' preferences for PHC services according to the interview guide. The interview guide was developed based on the current literature (see Appendix 1). The participants were asked about their perceptions and experiences about the advantages and disadvantages of PHC institutions compared with the secondary and tertiary hospitals, like “When you are sick, do you prefer to go directly to a secondary or tertiary hospital or a primary health care institution? What is the reason?” They were also asked if the PHC institutions met their needs, and the objective information of PHC institutions such as waiting time for visits, geographical location, etc. participants were encouraged to freely express their own ideas, and researchers made note of each interview.

After 50 interviews, we modified the interview guide to make our questions more accurate and appropriate. Limited time of the interviews continuously made in 2 PHCs did not provide any new themes, we considered it as the saturation of the interviews, and the interviewing would be ceased, followed by data analysis.

### Data Analysis

The framework method has been widely used in qualitative research on social policy related to medical issues and health care, and it provides clear steps to organize, process and produce highly structured outputs of summarized qualitative data (22). It was adopted for the management analysis of qualitative data in this study for two reasons. Firstly, this was a qualitative study to explore participants' preferences for PHC institutions, which aimed to provide policymakers with advice on the construction of PHC systems, and the thematic analysis method could be used to generate qualitative information that was useful for policymakers. Secondly, this approach was conducive to the management of large data sets (23). Considering the large amount of interview data involved in this study, the use of thematic analysis helped us to summarize key features of data on participants' preference as well as to highlight similarities and differences in data sets.

As data collection progressed, interview recordings were transcribed verbatim and cross-checked by two researchers. Contextual notes were recorded in the transcripts. The transcripts were then cleaned (removing redundancy) and organized by the sequence of interviews, while applied thematic analysis was used to identify the themes emerging from the data. Three members of our research team read the participants' interview transcripts to identify factors contributing to participants' preferences for PHC, which were then encoded to ensure accurate data coding and thematic construction. In addition, two other researchers conducted a detailed review of the above-encoded data and discussed any possible differences to achieve maximum consensus and rigor on the data coding. All the themes and sub-themes were dynamically checked when a new factor merged and updated if necessary by data coding, and eventually formulated with reference to literature work to generate a complete thematic framework that was integrated with all the identifiable factors contributing to participants' preferences for PHC. All the textual data was managed and retrieved using the NVIVO software (Version 10).

## Results

A total of 176 participants agreed to take part in the semi-structured interviews, and 142 finished the interviews, 34 of them failed to finish their interviews (dropout rate = 19.3%). The average age of the participants was 50 years old, ranging from 24 to 83, and 30.4% of them were male. The interviews mainly generated three key themes about contributing factors of patients' preferences for PHC: (1) attributes of primary healthcare services, (2) attributes of doctor workforce in primary healthcare, and (3) attributes of primary healthcare facility infrastructure. For each theme, there were several sub-themes (see Table 1).

**Table 1:** Themes and sub-themes of factors contributing to patients' preferences for PHC institutions.

**Theme**	**Sub-theme**	**Impact**	
		**Positive**	**Negative**
Section Attributes of primary healthcare services	Section Accessibility	+	
	Section Consultation time	+	
	Section Drug cost	+	
	Section Continuity of care	+	
	Section Referral system	+	
	Section Opening hours		–
	Section Waiting time		–
	Section Drug availability		–
Section Attributes of primary healthcare doctors' workforce	Section Doctors' attitude	+	
	Section Doctors' ability		–
	Section Doctor availability		–
Section Attributes of primary healthcare facility infrastructure	Section Basic facilities		–
	Section Diagnostic facilities		–
	Section Department setting		–

For each sub-theme, the participants reported whether it had positive impacts or negative impacts on the patients' preferences for PHC service.

### Attributes of PHC Services

There were eight attributes regarding the PHC services, and five of which had positive impacts on the choice of PHC institutions (Accessibility, Consultation time, Drug cost, Continuity of care, and Referral System), and the remaining three having negative impacts (Opening hours, Waiting time, and Drug accessibility in terms of choice and consistency in supply).

#### Accessibility (Positive Impact)

The accessibility of primary healthcare services mainly referred to geographical accessibility, i.e., the shortest distance from patients' home to PHC institutions. The interview results showed that it took <20 min for residents traveling from their community to the nearest PHC facility, which was considered convenient for them to seek for PHC. Participants also indicated an appropriate geographical distribution of health resources was important. They wanted to get access to PHC instantly when they needed it, which was described as:

“*About five bus stations, you can arrive at primary health care institution within a quarter of an hour” (Female, 59 years old)*

“*Actually, it (PHC institution) is much closer than other health care institutions.” (Male, 41 years old)*

#### Consultation Time (Positive Impact)

The time length of consultation between patients and doctors was also a very important factor for their preference for PHC. Sufficient consultation time made effective communications and ensured that all information related to the disease treatment (including treatment options and potential adverse reaction) could be achieved. While short consultation time hindered the patient's confidence in active participation in treatment. In general, most participants were satisfied with consultation time in PHC, which was described as:

“*Patients are less in PHC than those in tertiary hospitals, so doctors may ask patients more carefully, it is also good for his diagnosis” (Male, 44 years old)*

#### Drug Cost (Positive Impact)

Drug cost was one of the important factors affecting patients' preference. Since the costs of drug in PHC institutions were much lower than those in the secondary and tertiary hospitals, patients preferred to choose primary healthcare services, which were described as:

“*It is good enough for me. Drug costs here are lower than large hospitals. Reimbursement ratio is 85% here, but it is 75% in large (tertiary) hospitals. Anyway, it is 10% lower.” (Female, 59 years old)*

“*I get the prescription there (In tertiary hospitals) and buy the medicines back there in primary care facilities, just because the reimbursement ratio is higher there.” (Female, 34 years old)*

#### Continuity of Care (Positive Impact)

Participants said that the continuity of health care was very important because they needed primary care doctors to coordinate their health care, help them contact specialists, etc. They wanted to keep in touch with doctors so that doctors could fully understand their physical conditions. Participants reported that PHC institutions performed well in terms of continuity of care, and they were familiar with physicians or nurses in the PHC and preferred to choose PHC institutions because these acquaintances could make them feel secured, which was described as:

“*My family doctor will call me and ask me about my recent physical condition. He is very familiar with my illness” (Female, 59 years old)*

“*Yes, they sometimes come to our community. If the residents have any problems, they can consult him in the community. We (Middle-aged people) are now free to move, so we don't need their home visits. I just use a quarter walking to the primary care.” (Female, 54 years old)*

“*I think primary doctors often provide health services for our community residents, they come over and check the blood pressure for us, maybe once a week.” (Female, 49 years old)*

#### Referral System (Positive Impact)

Most participants said that when health care providers in PHC institutions encountered “disease which is beyond their scope,” they quickly referred that patient to a higher-level hospital, which provided benefits to the patient that the referral avoided unnecessary financial and health loss. Common reasons for referral included a lack of necessary medical equipment and doctors' diagnostic ability, or patients' request. The study results indicated that the referral efficiency of most PHC institutions in Nanjing was relatively high, and the referral process was quite smooth. It was described as:

“*If they (PHC institutions) are unable to diagnose, we will be referred to the secondary or tertiary hospitals, otherwise it will not be referred.” (Men, 59 years old)*

“*It's okay that you don't use the referral system, or otherwise you have to go through the Outpatient. Outpatient services are conveniently available in any hospitals, but its costs are generally higher than the costs of Consolidated Outpatient. But if you want to use it, you have to go through the Consolidated Outpatient at first place.” (Male, 66 years old)*

#### Opening Hours (Negative Impact)

The participants said that PHC institutions did not provide health services at night. If the patient suddenly felt sick and needed medication, he or she could only go to the emergency department of a tertiary hospital. This situation resulted in patients' sense of convenience of PHC, thus weakening the patient's preferences for PHC services, which were described as:

“*In theory, PHC institutions shall open at night, but no one is on duty at night in the primary care in fact, patients can't get medical treatment ……Because we don't have access to doctors there” (Female, 35 years old)*

#### Waiting Time (Negative Impact)

The waiting time was also very important for patients' preferences. A long waiting time could easily affect a patient's emotional status. While waiting time at PHC was usually much shorter than that at secondary or tertiary hospitals, patients were still dissatisfied with the waiting time at PHC. Most participants thought that 15 min was the longest acceptable waiting time at the PHC facilities, and patient's dissatisfaction grew if the waiting time exceeded 15 min. The patients hoped to see the doctor soon after the registration, but this expectation was hardly realized. In addition, different patients had different levels of tolerance for waiting time. For non-urgent patients, waiting time within half an hour was considered acceptable, while others who were busier showed less tolerance. The interviewees described it as:

“*Once I waited in line for 12 minutes, I was very… very anxious…It took more than an hour totally…… but there was no way, since there were so many people, everyone was queuing here.” (Female, 39 years old)*

“*There are a lot of patients here (PHC institution) and sometimes we may wait for a long time” (Female, 39 years old)*

#### Drug Accessibility (Negative Impact)

Most of the participants indicated that there was a shortage of medicines in PHC institutions, including the shortages of alternative drugs or pharmaceutical dosage forms. The shortage of drugs delayed the treatment of diseases and caused complaints and dissatisfaction of patients. In order to meet the patients' own needs for specific medicines, patients were referred to tertiary hospitals, which led to the reduced number of visits to PHC institutions. In addition, the lack of drugs increased the financial burden on patients, because the shortage of low-priced drugs led to more prescription of high-priced alternative drugs, or they referred to other health care institutions which might increase the patient's transport costs. Therefore, inaccessibility of medicines had a negative impact on patients' preferences for PHC. It was described as:

“*There are only some medicines for treating common diseases. The doctor said that the type of medicines is not determined by the community hospitals but the health department” (Male, 49 years old)*.

“*There is not enough kind of Chinese medicine here, so sometimes the doctor will prescribe for us to go to the pharmacy” (Female, 56 years old)*.

“*There is often a shortage of medicines here, sometimes I need to go there (PHC institution) twice to buy a medicine. Anyway, many people often complain about the lack of medicine. The doctor let us come back in a few days” (Female, 59 years old)*.

### Attributes of PHC Doctors' Workforce

Three factors about primary healthcare doctor workforce contributed to patients' preferences for PHC service: Doctors' attitude as positive impact; Doctors' ability and Doctor Accessibility as the opposite.

#### Doctors' Attitude (Positive Impact)

Participants believed that the doctors' attitudes toward them were a key factor affecting their preference for PHC, and they assumed that the doctors should listen to their description on symptoms and concerns. Most of the participants said that the PHC physicians were kind and friendly. They listened carefully for the needs of patients and take patients' opinions seriously. Patients were satisfied with these aspects of the PHC institution, which was described as:

“*The doctors here are much better… While they (doctors in tertiary hospitals) are more perfunctory” (Female, 83 years old)*

“*I am very satisfied with them because they have a good attitude” (Male, 80 years old)*

“*…but I like their good service attitude… and they are very considerate” (Female, 58 years old)*

“*If the doctors are not willing to treat us patiently in tertiary hospitals, I prefer to choose a doctor here (PHC institutions)” (Female, 36 years old)*

“*I think doctors there (tertiary hospitals) are not as good as those in community hospitals. They have no time to answer patients' questions and only let us take medical examinations” (Male, 62 years old)*

#### Doctors' Competence (Negative Impact)

The interviews suggested that the main reason for residents not to choose PHC was that the doctor's diagnostic ability and experience were perceived insufficient in PHC institutions. The participants said that they first considered the doctor's capabilities when having to decide between PHC service and secondary/tertiary hospital service, including the ability to cure the patients' disease, help patients understand their health status, and explain their treatment options. Unfortunately, most of the participants felt that PHC doctors did not fully possess this ability, such as:

“*You have to go to a tertiary hospital to check it out. This is undeniable. Doctors in tertiary hospitals can cure my disease while doctors here (PHC institutions) are definitely unable to cure my disease.” (Male, 66 years old)*

“*The doctor here (PHC facility) can cure a mild disease, as for serious disease, well, I'm not sure” (Male, 66 years old)*

“*The physicians' professional abilities are limited here (PHC institution), they can only treat some minor diseases, like common cold, I think” (Female, 25 years old)*

“*Anyway, I am not willing to see a doctor here. I just have an intravenous drip” (Female, 83 years old,)*

“*To be honest, it's impossible for a general practitioner to be proficient in all diseases, so they only need to understand the treatment of general diseases, but they shouldn't understand nothing” (Female, 83 years old)*

“*Actually, I originally wanted to have a family doctor, but I don't know how their abilities are and if they can cure common diseases.” (Male, 60 years old)*

#### Doctor Accessibility (Negative Impact)

The construction of family doctor system in China was still immature. The major problem was that the number of family doctors in PHC institutions was insufficient, so residents in some areas could not get access to family doctors. Patients, especially those with chronic diseases, had a great demand for family doctor services, the goal of assigning a family doctor for each family had not been achieved, which led to the low accessibility of family doctors in China. It was described as:

“*The family doctor system in our country is still in the verbal.…. In fact, I've never accessed to a real family doctor” (Male, 45 years old)*

### Attributes of Primary Healthcare Facility Infrastructure

Three factors about the infrastructure of healthcare facilities contributed to patients' preferences for PHC service, including basic facilities, diagnostic facilities, and department setting, all of which have a negative impact.

#### Basic Facilities (Negative Impact)

The environment in the waiting room was important for the patient because a comfortable environment could help alleviate the anxiety of the patient and the suffering caused by the disease. Participants expressed the hopes that the PHC's waiting room and clinic transfusion room would become more spacious and more users-friendly. They also hoped that PHC institutions provided comfortable seats and some reading materials of health education which could improve the patient comfort in the PHC and willingness to visit the PHC:

“*I think the environment here is ok…… if the space is bigger, it will be better” (59 years old, Female)*

“*You know what, the infusion room here is too small. The air condition worsens when the number of patients increase which aggravate the patients' discomfort. I think it's a big problem… I think most infusion rooms in primary care facilities are small” (Male, 59 years old)*

#### Diagnostic Facilities (Negative Impact)

During the interview, participants generally indicated that the diagnostic facilities of the PHC institutions could not fully meet their needs. The shortage of medical equipment directly restricted the realization of PHC institutions' functions and became the important reasons why patients did not choose PHC institution for medical treatment. The patient indicated that many diagnoses could not be carried out due to the lack of corresponding medical equipment, which directly hampered the doctor's diagnosis and weakened patients' confidence of visiting the PHC institutions. It was described as:

“*The tertiary hospitals have good medical conditions and more medical equipment than primary hospitals” (Female, 83 years old)*

“*For example, if a patient needs to have a coronary CT (computed tomography) or UCG (ultrasonic cardiogram), the primary care facility does not have these devices” (Male, 38 years old)*

“*There are too few medical devices here (PHC institutions) to make it impossible to diagnose serious diseases, so we don't prefer to come here” (Male, 66 years old)*

“*The large medical equipment of the PHC organization cannot be compared with the tertiary hospitals” (Female, 25 years old)*

“*The pediatricians' diagnosis and treatment techniques are not as good as those of tertiary hospitals, but we need to line up for a long time in tertiary hospitals” (Female, 38 years old)*

#### Department Settings (Negative Impact)

Participants indicated that the clinical department setting of PHC institution was still incomplete, which affected the provision of certain health care services. For example, some PHC institutions did not have the E.N.T. department, surgical department, stomatology department, etc. To some extent, the lack of medical departments had caused inconvenience to patients, which was described as:

“*I think the setting of the primary care facilities' department should be complete, just like those in tertiary hospital” (Female, 35 years old)*

“*The layout of the medical department here is not comprehensive, there is no stomatology department. You know what, I have to go to a specialist hospital to see oral diseases.” (Female, 41 years old)*

“*There is no pediatrics department here, so I will take him to a tertiary hospital if my kid is ill…But I think the community hospital should have pediatrics. Otherwise, it is more troublesome for us.” (Female, 37 years old)*

## Discussion

From a broader perspective, the patients' perception of PHC is affected by their trust with PHC. Usually, they believe that the PHCs were “generally inferior” and should only be used if the “patients-judged” problem was minor, rather than that they were actually “superior” for some situations. This study contributed by exploring in more detail about underlining factors that influence patients' preference and attitudes toward PHC. Through qualitative interviews, this study comprehensively explored the factors that contributing to patients' preferences for PHC in China. The results of this study showed that there were both positive and negative factors that affected Chinese patients' preferences for PHC, which needs further discussion as below.

For positive factors, this study indicated that Accessibility of primary healthcare services, Consultation time, Drug cost, Continuity of care, Referral System, and Doctors' attitude were the main positive influencing factors for preference for PHC, which should be strengthened to increase the PHC utilization. Firstly, this qualitative result showed that most patients were satisfied with the geographical accessibility of PHC. Previous studies suggested that the geographic accessibility of PHC institutions reflected the convenience of arriving at health care institutions, and determined whether residents could obtain the required health services timely to a certain extent, which in turn affected their preference for PHC services (24). Therefore, PHC institutions should be located considering their distance from each community under its coverage, ensuring that most residents can arrive within 15 min' walk (25).

Secondly, the participants attached great importance to the continuity of care in PHC institutions. Continuity of care means they do not have to repeat their medical history and personal background, which makes them feel secured, trustful, and comfortable to receive treatment. In China, the continuity of care depends on the PHC network and referral strategies of PHC institutions. Patients in the referral system can receive care from higher-level health care institutions. An effective referral system is strictly based on a clear classification of diseases (26). In order to encourage Chinese patients to use PHC as their first choice, it is recommended that the PHC institution should improve the construction of the referral system. The primary care doctor should make a reasonable referral according to the type and severity of the patients' disease. This requires refining the implementation norms of referral and developing a uniform referral standard between different regions. Specifically, an interconnected referral information system should be established between PHC institutions and other hospitals to ensure the operability of dual referral. Good information continuity helps improve the efficiency, safety and quality of the service.

Thirdly, regarding doctors' attitude, almost all participants indicated that primary care doctors should respect their patients, be kind to their patients, and leave enough time for patients to consult. If patients are dissatisfied with doctors' attitudes or feel restricted in speaking openly, they will not seek help at PHC institutions (27). In this study, the regulations at some PHC institutions stipulated that doctors should keep the diagnosis procedure for at least 20 min to ensure that doctors had enough time to explain the results of diagnosis and their options on the treatment during the consultations with their patients. This also allowed time for patients to give feedbacks and ask questions. Long consultation time with a good attitude of doctors will certainly increase patients' preferences for PHC institutions.

Besides the positive factors, there are some negative factors that demand more attention. Firstly, among the sub-themes of primary healthcare services, drug accessibility should be raised much concern. The drug accessibility greatly affected patients' preferences for PHC institutions. In China, the shortage of medicine in PHC institutions is very common, especially for essential medicines. Usually, these are less-demanded or low-priced clinically essential drugs that have the same effect on a certain disease (28). Due to the low price and small demand of such drugs, pharmaceutical manufacturers make fewer profits in these products than others, so they reduce or stop the production, which aggravates the undersupply of drugs. PHC institutions in the same region should work together to build a network platform for drug storage and supply information so that the production, circulation, utilization, and supervision of drugs can be flexibly arranged among the regional PHC network. This platform can improve communication efficiently, feedback information timely, and find/solve problems accurately. At the same time, PHC institutions should regularly monitor drug inventory information, release warning information on shortage drugs timely, strengthen communication with drug manufacturers and health departments to prevent drug shortages (29).

Secondly, in terms of primary healthcare doctor workforce, doctors' accessibility and patients' perceived ability have big effects on patients' preference for PHC. In the current health system in China, many residents cannot access family doctors due to the limited number of family doctors at PHC institutions. Because of differences in social and economic development across the country, the provision and quality of family doctor services is uneven among the cities or even districts within one city. The possible reason is that the training mechanism of family doctors in China is still not perfect, leading to shortage of family doctors and their uneven service capacity. Another reason could be the lack of basic public health service funds for PHC institutions (such as medical insurance funds) to support the implementation of the family doctor system (30). Therefore, the realization of the family doctor system requires the expansion of medical students in colleges, especially general practitioners, to enhance family doctors' workforce and competence. Government should also take advantage of incentives to encourage family doctors to work at primary care facilities. At the same time, a family doctor's treatment expenditure should be included in the medical insurance, which provides partial compensation for the medical and medicine expenses of patients with chronic diseases (31).

In addition, the current diagnosis and treatment ability of doctors in PHC institutions is low, making it very difficult to meet the needs of patients selecting PHC services. Such a phenomenon is mainly due to the poor salary of primary care doctors, which makes it difficult to recruit high-quality doctors. Studies have shown that the average income of primary care doctors in China is lower than that of tertiary hospitals, which is different from most developed countries (32). Developed countries usually attract and stabilize primary health care workers with high salary incentives. The low income of primary care doctors should be given enough attention. It is suggested that PHC institutions formulate a reasonable and perfect performance appraisal system based on the opinions of medical workers on the construction of PHC system (33). In particular, it needs to increase the basic salary of primary medical staff substantially (34). Additionally, it suggests an enhancement in skill training and comprehensive competence development of in-patient health care providers by offering the providers with opportunities of advanced studies in tertiary hospitals. It also suggests the improvement of healthcare provider training system to cultivate primary providers with higher competence, which may help covering the deficiency of care provision by PHC institutions. Spontaneously, the Chinese government should promote the health provider training programs in higher education system by reinforcing the training and discipline construction fitting the standard of providers in PHC institutions.

Thirdly, in terms of the healthcare facilities infrastructure, diagnostic facilities have non-negligible effects on patients' preference for PHC. It was found that the lack of medical equipment in PHC institutions hinders the utilization of PHC by patients. Since the quantity and quality of medical equipment in primary care facilities are lower than those in hospitals, which results in poor accuracy of diagnosis results, patients do not prefer PHC institutions. The fundamental reason for this phenomenon is that current PHC institutions are underfunded, which makes certain difficulties in the allocation of medical resources in China. For a long time, the sales revenue of Chinese health care institutions had been the main source of income. However, in 2009, the Chinese health department issued a “zero-markup” drug policy, which forbad the drug addition of PHC institutions. To a certain extent, this policy has caused a shortage of operating funds for PHC institutions and lessened their ability to purchase medical equipment (35). In light of the shortage of equipment in PHC institutions, we draw on the successful experiences of other countries to improve the situation. From the experience of Japan, the government pays attention to attract various social resources and guarantee the supply of medical services in the policy, while enjoying more autonomy in operation management. Although China has introduced a number of policies to encourage social funds to run medical services in 2015, it is still in its infancy, and the implementation still needs to be improved (36, 37). Germany has large diagnostic equipment in hospitals among different regions, and these clinics can share the laboratory and auxiliary inspection equipment together (38). Based on the experience of the above countries, it is suggested that the Chinese government should implement private capital investment in medical institute to optimize the funding structure of PHC institutions (39).

## Limitation

To our knowledge, this is the first qualitative study that explores contributing factors to patients' preferences for PHC institutions in China. However, this study has some limitations that can be addressed in future studies. First, this study focused on Nanjing City, where PHC services are relatively complete compared with less developed cities. Thus, future studies can be extended to other areas with less developed health systems, such as the western regions of China. Second, since this is a qualitative research, our results only indicate the reasons why patients do not prefer PHC institutions. We do not know the extent to which these reasons impact. Therefore, future researches can use quantitative design to explore the influencing degree of these factors on patients' preferences for PHC services. Third, we only collected the information from patients in this study. Future studies can collect information from other key stakeholders of PHC, such as PHC staffs and officials of health department.

## Conclusion

Chinese patients' preferences of PHC institutions can be affected by three types of factors. To increase patients' preferences and utilization of PHC service, policy interventions are needed to improve doctor workforce and infrastructure of PHC institutions.

## Data Availability Statement

The datasets generated for this study are available on request to the corresponding author.

## Ethics Statement

The studies involving human participants were reviewed and approved by the Ethics Committee of the China Pharmaceutical University (Project number: CPU2018016). The patients/participants provided their written informed consent to participate in this study.

## Author Contributions

XX and HH designed and planned the study and have been responsible for critical revision of the manuscript for important intellectual content. WZ, GL, JL, and WL performed all the patient interviews, evaluated, and scored the transcribed interviews. WZ and JL drafted and wrote the manuscript. XX, CU, and JL have actively participated in the design of the qualitative part of the study and in writing the manuscript. All authors contributed to the article and approved the submitted version.

## Conflict of Interest

The authors declare that the research was conducted in the absence of any commercial or financial relationships that could be construed as a potential conflict of interest.

## Abbreviations

PHC: Primary Health Care
NHMS: National Hierarchical Medical System
PDGL: Primary Diagnosis at Grassroots Level
CT: Computed Tomography
UCG: Ultrasonic Cardiogram
UHC: Universal Health Coverage.
