# Supplementary material for: Factors Contributing to Patients' Preferences for Primary Health Care Institutions in China: A Qualitative Study
Source: Front Public Health. 2020 Aug 18;8:414. doi: 10.3389/fpubh.2020.00414 (PMC7461976; doi:10.3389/fpubh.2020.00414)
Supplement: Supplementary file 1 [file Data_Sheet_1.DOCX]

# Appendix1- Interview guide

1. When you were not feeling well before, did you skip the primary health care institution and choose other medical institution?

*Prompt when necessary: What kind of disease? Which hospital to choose?*

1. Do you prefer to go to a community health service or not? Why ?

*Prompt when necessary*：

- Type of disease (general (cold fever), chronic disease (hyperlipidemia, diabetes), severe (intestinal colic))
- Recommended by family or friends
- Treatment effect, quality of service (whether misdiagnosed, too much prescription)
- Your own preferences
- Have a familiar doctor (how to know)
- Doctor's experience/diagnostic ability (whether it meets your expectations)
- had an unpleasant experience in a community hospital
- The environment of the waiting room / consulting room
- Advanced and comprehensive medical equipment
- Medical insurance restrictions
- Medical records are recorded here(continuity)

1. What is the reason for choosing a community hospital for this visit? What do you think the community hospital needs to improve?
2. What services does the primary health care institution provide? Can these services meet your needs?

Tips when necessary: diagnosis, purchase of medicine, health education, dentistry, optometry, surgery, chronic disease management

1. How long does it take from your home to primary health care institution? Is the transportation convenient?

How long is the waiting time for registration? What do you think of this length of time?

How long does the doctor's treatment take? What do you think of this length of time?

1. Is the doctor's judgment of your condition at the primary health care institution accurate? Do you have confidence in the physicians’ diagnostical capability?
2. What do you think about the cost of medical treatment in primary health care institution? How much does it cost you to see a doctor in a community hospital every year? How much can your health insurance reimburse?
3. Overall, are you satisfied with the services provided by primary health care institution?

If yes: Please give two examples. What are you most satisfied with?

If no: What is your most dissatisfied? What suggestions do you have for improvement?

**Thank you for participating in this interview. Do you have any other ideas related to this topic?**

**Age** : **_____**

**Gender:** □Male □Female

**Education:** Elementary school or lower\Junior high school、High school、Junior college、Undergraduate、Graduate student or higher

**Occupation:** Public servants、Teacher、Manager、Worker、Farmer、Individual household、Others、None
